# Comparison of methods to assess onset of breast development in the LEGACY Girls Study: methodological considerations for studies of breast cancer

**DOI:** 10.1186/s13058-018-0943-9

**Published:** 2018-04-18

**Authors:** Lauren C. Houghton, Julia A. Knight, Mary Jane De Souza, Mandy Goldberg, Melissa L. White, Karen O’Toole, Wendy K. Chung, Angela R. Bradbury, Mary B. Daly, Irene L. Andrulis, Esther M. John, Saundra S. Buys, Mary Beth Terry

**Affiliations:** 1 Department of Epidemiology, Columbia University Mailman School of Public Health, 722 West 168th Street, New York, NY 10032 USA; 2 Lunenfeld-Tanenbaum Research Institute, Mount Sinai Hospital, Toronto, ON Canada; 3 Division of Epidemiology, Dalla Lana School of Public Health, University of Toronto, Toronto, ON Canada; 4 Kinesiology and Physiology, College of Health and Human Development, The Pennsylvania State University, University Park, PA USA; 5 Department of Medicine, Huntsman Cancer Institute, University of Utah Health Sciences Center, Salt Lake City, UT USA; 6 Herbert Irving Comprehensive Cancer Center, Columbia University Medical Center, New York, NY USA; 7 Department of Pediatrics, Columbia University Medical Center, New York, NY USA; 8 Department of Medicine, Columbia University Medical Center, New York, NY USA; 9 Department of Medicine, Perelman School of Medicine, University of Pennsylvania, Philadelphia, PA USA; 10 Department of Medicine, Hematology/Oncology, Perelman School of Medicine, University of Pennsylvania, Philadelphia, PA USA; 11 Medical Ethics and Health Policy, Perelman School of Medicine, University of Pennsylvania, Philadelphia, PA USA; 12 Department of Clinical Genetics, Fox Chase Cancer Center, Philadelphia, PA USA; 13 Department of Molecular Genetics, University of Toronto, Toronto, ON Canada; 14 Cancer Prevention Institute of California, Fremont, CA USA; 15 Division of Epidemiology, Department of Health Research and Policy, Stanford University School of Medicine, Stanford, CA USA; 16 Stanford Cancer Institute, Stanford University School of Medicine, Stanford, CA USA

**Keywords:** Breast development, Breast cancer, Measurement, Puberty

## Abstract

**Background:**

Younger age at onset of breast development, which has been declining in recent decades, is associated with increased breast cancer risk independent of age at menarche. Given the need to study the drivers of these trends, it is essential to validate methods to assess breast onset that can be used in large-scale studies when direct clinical assessment of breast onset is not feasible.

**Methods:**

Breast development is usually measured by Tanner stages (TSs), assessed either by physical examination or by mother’s report using a picture-based Sexual Maturation Scale (SMS). As an alternative, a mother-reported Pubertal Development Scale (PDS) without pictures has been used in some studies. We compared agreement of SMS and PDS with each other (*n* = 1022) and the accuracy of PDS with clinical TS as a gold standard for the subset of girls with this measure (*n* = 282) using the LEGACY cohort. We further compared prediction of breast onset using ROC curves and tested whether adding urinary estrone 1-glucuronide (E1G) improved the AUC.

**Results:**

The agreement of PDS with SMS was high (kappa = 0.80). The sensitivity of PDS vs clinical TS was 86.6%. The AUCs for PDS alone and SMS alone were 0.88 and 0.79, respectively. Including E1G concentrations improved the AUC for both methods (0.91 and 0.86 for PDS and SMS, respectively).

**Conclusions:**

The PDS without pictures is a highly accurate, sensitive, and specific method for assessing breast onset, especially in settings where clinical TS is not feasible. In addition, it is comparable to SMS methods with pictures and thus easier to implement in large-scale studies, particularly phone-based interviews where pictures may not be available. Urinary E1G can improve accuracy over than PDS or SMS alone.

**Electronic supplementary material:**

The online version of this article (10.1186/s13058-018-0943-9) contains supplementary material, which is available to authorized users.

## Background

Puberty is an important stage of development that impacts future breast cancer risk [1]. Recent increases in the incidence of early-onset invasive breast cancer [2] may be related to recent declines in the age at initiation of breast development [3]. Earlier age at breast onset, independent of age at menarche, which unlike age at breast development has been more stable in recent decades, has been found to be associated with an increased risk for breast cancer [4]. Large-scale epidemiologic studies investigating factors that may explain risk of early breast onset are needed across diverse populations. Therefore, it is important to use methods that can assess the timing of breast onset that are both sensitive and specific as well as feasible in large-population studies. Because timing of breast onset has been shown to vary by ethnicity and obesity [5–7], it is also important to evaluate whether different methods to assess the onset of breast development vary by these factors.

Breast development can be measured by physical examination by clinicians or through guardian report or self-report based on questionnaires, either with or without picture prompts. Clinicians perform a visual and physical assessment, sometimes with palpation, of breast development according to Tanner stages (TSs) established in 1969 [8]. Although clinician assessment via physical examination is considered the gold standard [9, 10], self-reports and guardian reports are often used in lieu of the physical examination, especially in large-scale epidemiological studies in which conducting physical examinations is often not feasible. One such method is the Sexual Maturation Scale (SMS), a questionnaire-based tool that asks respondents to rate breast development based on pictures that correspond with the five TSs [11]. Another commonly used questionnaire-based instrument is the Pubertal Development Scale (PDS) [12]. A key advantage of the PDS is that it is question-based and does not involve pictures and can therefore be queried over the phone and/or more easily included in questionnaires.

Given the need to have scalable methods that accurately reflect pubertal development stage, the purpose of the present study was to compare the SMS and PDS with clinical TS to assess the specificity and sensitivity of reported measures of breast onset. Given that estrogens lead to increased epithelial proliferation in terminal end buds of the mammary gland, resulting in the onset of breast development [13], a secondary aim of this study was to evaluate whether the assessment of hormonal measures in premenarcheal girls increases the validity of guardian-reported methods of breast onset. Estrone 1-glucuronide (E1G), an estrogen metabolite in urine, is an indicator of total circulating estrogens, which rise before puberty [14]. Previous studies have found both the PDS and SMS to be accurate measures of breast development [15–18], but none have simultaneously compared SMS, PDS, and hormonal biomarkers with clinical TS.

## Methods

### Study population

The LEGACY Girls Study is a five-site study of pubertal development in 1040 girls ages 6–13 years at recruitment, half of whom have a family history of breast cancer (*for details, see* [19, 20]). Prior validation studies have not been conducted in cohorts enriched with individuals who have a family history of breast cancer. Because pubertal development measures are important to breast cancer risk, it is essential to evaluate whether differential measurement error exists based on breast cancer family history. Across all five study sites, classification of pubertal timing was based on the Growth and Development Questionnaire, which includes both the PDS [12] and SMS [11]. Mothers/guardians for girls of all ages and girls aged 10 years or older completed the questionnaire every 6 months. Because 97% of girls participated with their biological mother [19], we refer to them as the mother for the remainder of the paper. For the purpose of this study, we used the first available mother reports of breast onset.

### Tanner staging

TS assessment includes evaluation of the development of both breasts and areolas, with TS1 representing prepubertal development, TS2 representing the onset of breast development, TS3 representing further enlargement of breast and areola without separation of their contour, TS4 representing the areola and papilla forming a secondary mound above the level of the breast, and TS5 representing full breast maturity.

### Pubertal Development Scale

Using the PDS, mothers assessed breast development by responding to the question, “How far along is your daughter in the development of her breasts?” with five possible answer options: (1) has not yet started breast development, (2) barely started breast development, (3) breast development is definitely underway, (4) breast development seems complete, or (5) no answer. Option 2, “barely started,” corresponds with the onset of breast development and TS2 [12].

### Sexual Maturation Scale

The SMS instructions were as follows: “The drawings below show five different stages of breast development. A girl can go through each of the five stages, although some girls skip some stages. Look at each drawing, and read the description. Which of these drawings looks most like your daughter’s stage of development?” Mothers rated their daughter’s breast development by selecting one of five line drawings showing TS1–TS5 [11]. We collapsed stages 4 and 5 to convert the SMS 5-point scale into a 4-point scale to directly compare with the PDS. TS2 marks the onset of breast development.

### Clinical TS

At two study sites, trained research staff or a physician performed standardized clinical breast Tanner staging on 282 girls [21]. Three clinical raters from New York and one from Utah were trained concurrently on the determination of breast TS using visual inspection along with palpation when necessary. If it was difficult to distinguish between breast bud development (TS2) and fat tissue, then the breast was palpated with the girl’s permission, and a second score based on both visualization and palpation was recorded. As in the SMS, breast onset is marked by TS2. Clinician interrater reliability for Tanner breast stage was high, with weighted kappa scores ranging from 0.93 to 1.00 and kappa scores for T2+ vs T1 of 0.94–1.00 [21].

### Hormone measurement

Microtiter plate competitive enzyme immunoassays (EIAs) were used to measure E1G, which was assessed in a first-morning void provided by premenarcheal girls at the same clinical visit as when maternal PDS and SMS and clinical TS were reported. The E1G EIA uses a polyclonal capture antibody R522-2 from Coralie Munroe at the University of California (Davis, CA, USA). The competitor for this assay is E1G conjugated to horseradish peroxidase [22]. An endpoint substrate color reaction is developed with azino-bis-ethylbenzthiazoline sulfonic acid and peroxidase. E1G standards from Sigma-Aldrich (St. Louis, MO, USA) were used for the standard curves, and high and low internal controls were in-house samples. The interassay coefficients of variation for high and low internal controls for the E1G assay were 14.7% and 13.1%, respectively. The sensitivity of the E1G assay was 5.2 ng/ml. All urine samples were corrected for specific gravity using a hand refractometer (NSG Precision Cells, Inc., Farmingdale, NY, USA), and the concentration determined from the assay was divided by the specific gravity to correct for hydration status [23–26].

### Additional covariates

At each study visit, height and weight were measured twice by trained research staff. Averaging the two measures, we calculated age-specific height, weight, and body mass index (BMI) percentiles based on Centers for Disease Control and Prevention (CDC) growth charts [27]. Girls were classified as overweight if their BMI was equal to or above the 85th percentile. Girls were classified as having a family history of breast cancer if the participating mother reported a breast cancer family history in the daughter’s first- or second-degree relatives.

### Statistical models

We calculated percent agreement and kappa statistics between the first available mother’s report of breast onset using the question-only PDS and the picture-based SMS. We also assessed the sensitivity and specificity of PDS using the clinical TS as the gold standard. To examine whether mother’s reports for some subgroups of girls were less accurate, we assessed whether age, breast cancer family history, BMI, or race/ethnicity influenced the accuracy of the mother’s classification. We generated ROC curves to compare the PDS and SMS with clinical TS. We compared assessment of breast onset in two ways: we compared T2 with T1 and T2–T5 (T2+) with T1. We tested if adding other covariates improved the AUC of these models. Using Weibull models, we compared the median age at breast onset derived from the two mother’s report methods (PDS and SMS) with the median age from the clinical TS in a subset of girls who had all three measures at baseline (*n* = 200). We also compared the different pubertal staging methods with first morning urinary E1G concentrations that were collected at the same clinical visit as the PDS, SMS, and clinical TS. We used linear regression to compare E1G, after logarithmic transformation, at each stage of breast development as reported by mothers using the PDS and SMS. Data are reported as the mean and 95% CI with SEM.

## Results

### PDS vs SMS

In the overall cohort (*N* = 1022 with complete information; see Additional file 1: Table S1), PDS and SMS reports of breast onset stage were in high agreement (89.6%) (Table 1). The overall weighted kappa value was 0.80. The weighted kappa value was higher for reports from mothers of girls younger than 10 years old than from mothers of girls aged 10 years or older (Table 1).

**Table 1:** Agreement between Pubertal Development Scale and Sexual Maturation Scale methods of assessing breast onset, by sample characteristics in the LEGACY Girls Study

Description	PDS^a^ vs SMS^b^			
	2+ vs 1			
	No. of subjects	Agreement	Kappa value	95% CI
All ages combined	1022	89.6	0.8	0.7–0.9
Daughter aged < 10 years	500	92.4	0.7	0.6–0.8
Daughter aged ≥ 10 years	522	87	0.5	0.4–0.6
Family history				
Positive	529	90.9	0.8	0.7–0.9
Negative	493	88.2	0.8	0.7–0.9
BMI percentile^c^				
BMI ≥ 85th percentile	188	90.4	0.8	0.7–0.9
BMI < 85th percentile	815	89.6	0.8	0.7–0.9
Race/ethnicity				
White non-Hispanic	636	89.8	0.8	0.7–0.9
Hispanic	189	89.9	0.8	0.7–0.9
Other (black, Asian, other)	197	88.8	0.7	0.7–0.9
Site				
Philadelphia	152	92.3	0.7	0.6–0.8
New York	166	92.2	0.7	0.6–0.8
Utah	161	92.8	0.7	0.6–0.8
Ontario	191	93.3	0.8	0.7–0.9
California	352	91.2	0.7	0.6–0.8

*Abbreviations: BMI* Body mass index, *PDS* Pubertal Development Scale, *SMS* Sexual Maturation Scale

^a^PDS is a questionnaire-based assessment of breast development without pictures

^b^SMS is a questionnaire-based assessment of breast development that uses pictures to illustrate the five Tanner stages of breast development

^c^Missing BMI for 19 girls

### PDS vs clinical TS

PDS was also highly accurate in relation to clinical TS (87.9%) (Table 2). The overall weighted kappa value was 0.80, and sensitivity and specificity were 86.6% and 89.6%, respectively. Sensitivity was lower for reports from mothers of younger daughters than older daughters (62.5% vs 91.8%), but specificity was higher (95.8% vs 45%). Sensitivity was similar between mothers of girls with positive and negative family histories. Mothers of overweight girls (as defined on the CDC 85th percentile) had higher sensitivity (96.9% vs 83.0%) but lower specificity than mothers of nonoverweight girls (65.0% vs 93.1%).

**Table 2:** Accuracy of Pubertal Development Scale method of assessing breast onset compared with clinical Tanner scale, by sample characteristics in the LEGACY Girls Study

Description	PDS^a^ vs clinical TS^b^					
	2+ vs 1					
	No. of subjects	Accuracy (%)	Kappa value	95% CI	Sensitivity	Specificity
All ages combined	282	87.9	0.8	0.7–0.9	86.6	89.6
Daughter aged < 10 years	165	91.9	0.6	0.4–0.8	62.5	95.8
Daughter aged ≥ 10 years	117	83.8	0.4	0.2–0.6	91.8	45
Family history						
Positive	122	89.3	0.8	0.6–1.0	86.6	92.2
Negative	155	87.5	0.7	0.5–0.9	86.7	88
BMI percentile^c^						
BMI ≥ 85th percentile	50	84.6	0.7	0.4–0.9	96.9	65
BMI < 85th percentile	223	89.1	0.8	0.7–0.9	83	93.1
Race/ethnicity						
White non-Hispanic	189	87.9	0.7	0.6–0.8	100	100
Hispanic	60	90	0.8	0.5–1.1	84.8	89.9
Other (black, Asian, other)	33	87.9	0.8	0.5–1.1	81.3	94.1
Site						
New York	135	89.6	0.8	0.6–1.0	86.7	92
Utah	147	86.4	0.7	0.5–0.9	84.5	87.6

*Abbreviations: BMI* Body mass index, *PDS* Pubertal Development Scale, *TS* Tanner scale

^a^PDS is a questionnaire-based assessment of breast development without pictures

^b^Clinical TS is a physical examination of breast development and rated according to the five Tanner stages of breast development

^c^Missing BMI for 19 girls

### Hormone concentration by pubertal staging

Mean concentrations of E1G increased incrementally with each maturity stage as measured by PDS, SMS, or clinical TS (Table 3). Hormones were statistically higher in each subsequent stage for each assessment method. E1G concentrations were 0.80–0.88 ng/ml higher in girls rated at T2+ than girls in T1 across all measures. Girls with breast onset (T2+) according to any of the three different methods had E1G levels above 2 ng/ml, suggesting a possible hormonal threshold for breast onset.

**Table 3:** Mean urinary estrone 1-glucuronide levels and 95% CIs across stages of puberty, according to Pubertal Development Scale and Sexual Maturation Scale in the LEGACY Girls Study

Stage	PDS^a^				SMS^b^			Clinical TS^c^		
	No. of subjects	Mean (ng/ml)	95% CI		No. of subjects	Mean (ng/ml)	95% CI	No. of subjects	Mean (ng/ ml)	95% CI
1	96	1.25	1.11–1.39		116	1.34	1.21–1.47	103	1.29	1.16–1.43
2	36	1.91	1.51–2.32		20	2.07	1.61–2.54	36	1.96	1.56–2.36
3	21	2.34	1.87–2.81		13	2.30	1.76–2.83	11	2.31	1.74–2.89
4	0				4	2.76	1.92–3.59	3	3.94	1.98–3.87
2–4	57	2.07	1.70–2.44		37	2.23	1.83–2.62	50	2.10	1.72–2.47

*Abbreviations: PDS* Pubertal Development Scale, *SMS* Sexual Maturation Scale, *TS* Tanner scale

^a^PDS is a questionnaire-based assessment of breast development without pictures

^b^SMS is a questionnaire-based assessment of breast development that uses pictures to illustrate the five Tanner Stages of breast development

^c^Clinical TS is a physical examination of breast development and rated according to the five Tanner stages of breast development

**Fig. 1:**
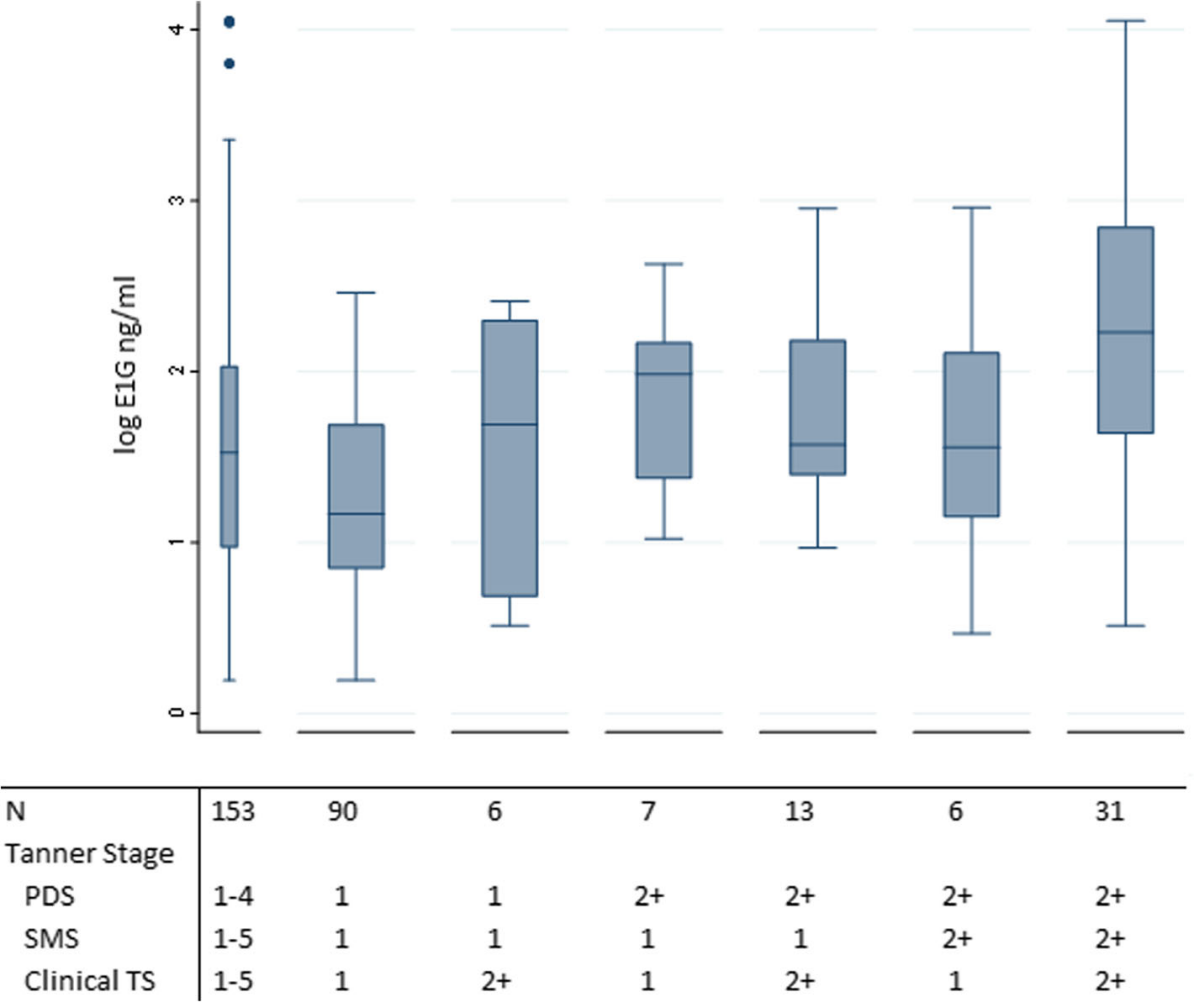
Estrone 1-glucuronide (E1G) concentrations according to concordant and discordant ratings of breast onset in the LEGACY Girls Study. Mean E1G values were 1.2 ng/ml in girls at T1 and concordant on all three measures (second box plot), 2.3 ng/ml in girls at stage T2+, and concordant on all three measures (last box plot). Mean E1G ranged from 1.5 to 1.8 ng/ml for girls with at least one discordant measure. *PDS* Pubertal Development Scale, *SMS* Sexual Maturation Scale

The distribution of E1G in the subset of girls (*n* = 153) with all three (PDS, SMS, and clinical TS) measures of breast development and a hormone measurement available is shown in Fig. 1. Mean E1G values were 1.2 ng/ml in girls at stage 1 and 2.3 ng/ml in girls at T2+ when all three methods of breast onset were concordant. Values ranged from 1.5 to 1.8 ng/ml for girls with at least one discordant measure.

Using clinical TS as the gold standard, the AUC (TS1 vs TS2+) for PDS alone was higher (0.88) than for SMS (0.78) alone (Table 4) or E1G alone (0.77). The AUCs for both PDS and SMS improved with the inclusion of hormone measurements (AUC = 0.91 and 0.86 for PDS and SMS, respectively). Adding additional predictors such as race/ethnicity, overweight status, and family history improved the AUC by 1% or less.

**Table 4:** Discrimination of the Pubertal Development Scale and Sexual Maturation Scale compared with clinical Tanner stage, as assessed by AUC in the LEGACY Girls Study

	Clinical Tanner stage (T1 vs T2+) AUC (*n* = 153)	Clinical Tanner stage (T1 vs T2) AUC (*n* = 153)
Model A		
PDS^a^	0.88	0.84
E1G (ng/ml)	0.91	0.87
Race/ethnicity	0.89	0.86
Height (% for age)	0.89	0.84
Weight (% for age)	0.88	0.84
Overweight (> 85th percentile BMI)	0.90	0.86
Family history	0.87	0.83
SMS^b^	0.78	0.68
E1G (ng/ml)	0.86	0.79
Race/ethnicity	0.81	0.70
Height (% for age)	0.78	0.68
Weight (% for age)	0.78	0.68
Overweight (≥ 85th percentile BMI)	0.80	0.69
Family history	0.78	0.68
Model B		
PDS^a^	0.88	0.84
+ E1G (ng/ml)	0.91	0.87
+ Race/ethnicity	0.91	0.88
+ Overweight (> 85th percentile BMI)	0.92	0.88
+ Family history	0.92	0.88
SMS^b^	0.78	0.68
+ E1G (ng/ml)	0.86	0.79
+ Race/ethnicity	0.86	0.80
+ Overweight (> 85th percentile BMI)	0.87	0.80
+ Family history	0.87	0.80
Model C		
PDS, SMS, E1G	0.91	0.87

*Abbreviations: BMI* Body mass index, E1G Estrone 1-glucuronide, *PDS* Pubertal Development Scale, *SMS* Sexual Maturation Scale

Model A: These models compare mother’s report (PDS or SMS) with clinical TS and add each covariate one at a at time

Model B: These models compare mother’s report (PDS or SMS) with clinical TS and are nested so that each subsequent covariate is added to the previous model

Model C: This model includes PDS, SMS, and E1G to test if the combination of all three measures yields a higher AUC than either PDS + E1G or SMS + E1G

^a^PDS is a questionnaire-based assessment of breast development without pictures

^b^SMS is a questionnaire-based assessment of breast development that uses pictures to illustrate the five Tanner Stages of breast development

### Impact on predicted median age at breast onset

**Fig. 2:**
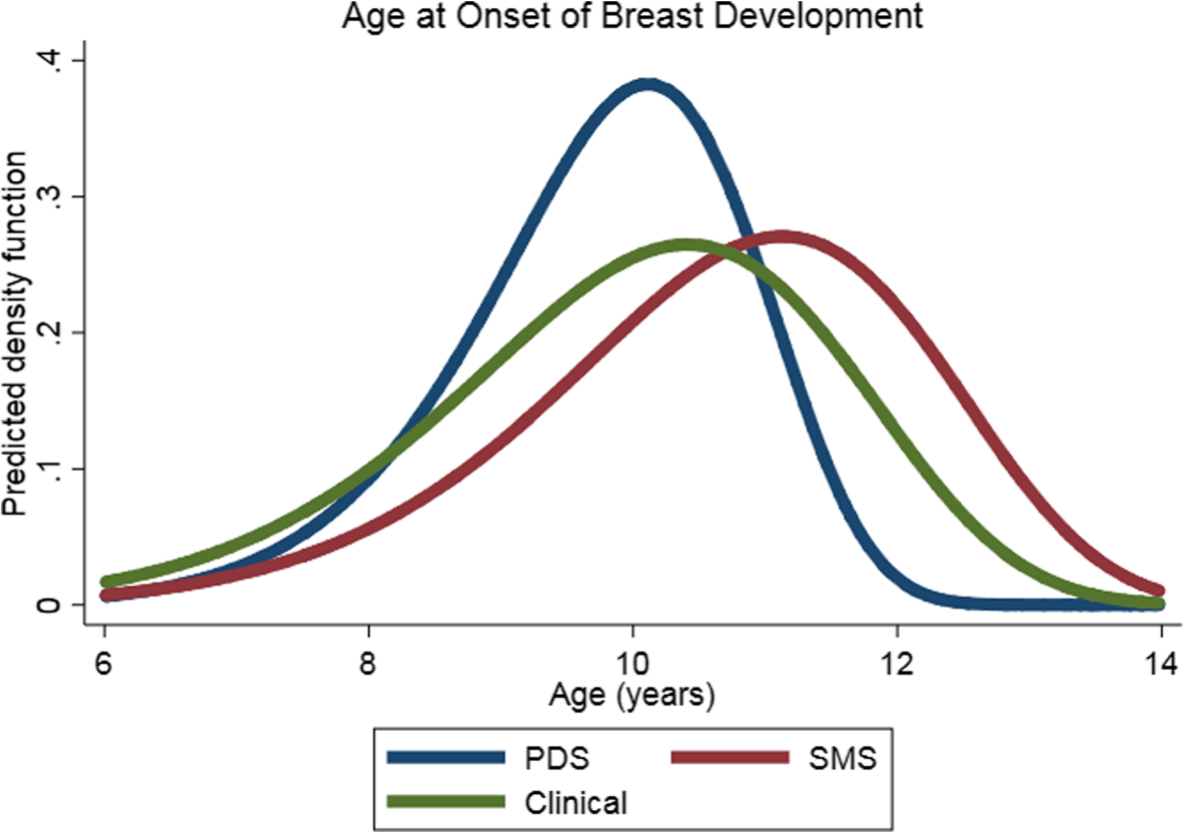
Median age at breast onset derived from unadjusted Weibull models, by pubertal assessment method in the LEGACY Girls Study (*n* = 200). The median ages in years (IQR) were 9.9 (9.1–10.5) using Pubertal Development Scale (PDS), 10.8 (9.7–11.8) using Sexual Maturation Scale (SMS), and 10.1 (9.0–11.1) using clinical Tanner stage (TS)

The median ages (IQR) at breast onset were 9.9 years (9.1–10.5), 10.8 (9.7–11.8), and 10.1 (9.0–11.1) as assessed by the PDS, SMS, and clinical TS, respectively (Fig. 2). This translates to PDS underestimating age at breast onset by 2.4 months and SMS overestimating it by 8.4 months.

## Discussion

Our results demonstrate that breast onset determined by mother’s report using the PDS corresponds well with both clinical TS (the existing gold standard) and the physiological changes in gonadal steroid hormone concentrations that drive pubertal maturation. Furthermore, the discrimination from mother’s report of breast onset using the PDS in predicting TS2+ was better than using mother’s report of SMS. Our findings also suggest that E1G in combination with mother’s report further improves the discrimination of breast onset. Although the majority of puberty studies use clinical TS to assess breast onset [28–33], large-scale studies of pubertal development using mother’s report without a clinical visit can produce accurate and valid measures of breast onset, particularly when additional hormone measures are added.

Mother’s report using PDS had higher discrimination, agreement, and accuracy than mother’s report using SMS. We previously published the kappa values and percent agreement between mother’s report using SMS with clinical TS in the same cohort and found agreement with clinical TS to be 73% compared with the 88% reported here with PDS [21]. Mothers were less accurate at identifying TS2+ when using SMS, perhaps because the pictures of TS2 capture size in addition to areolar development, leading mothers to downgrade their daughter’s breast development. However, the PDS questions preceded the SMS pictures, and this order of administration may have affected the comparison as well. The kappa value and percent accuracy of mother’s reports of breast onset using either the PDS or SMS compared with clinical TS were consistent and higher in our study than those previously reported in other studies of pubertal development [15–17]. Specifically, in a study consisting of 78 girls (aged 9–14 years), the kappa value between mother’s report using either PDS or SMS compared with clinical TS was 0.36 with 47–49% agreement [15]. The age range of girls in our study was wider (6–16 years) than in other studies, allowing us to assess differences in accuracy according to age. We observed that mothers of young daughters (< 10 years old) were accurate in reporting the absence of breast onset (specificity) and mothers of older daughters (≥ 10 years old) were accurate in reporting the presence of breast onset (sensitivity); thus, the wider age range and more mothers of young (6–9 years old) and older (14–16 years old) daughters in our cohort may explain the higher overall agreement in our study. Although we were unable to compare PDS and SMS with clinical TS in our full cohort, the sample size of the subset with all available measures was larger than that in any previous studies which have assessed mother’s reports of breast development [15–18]. Our present results and other recent results [15] differ from those of a 2002 review of pubertal assessment methods in which the authors concluded that the PDS was the least valid method compared with SMS and other methods. The studies that have shown the utility of PDS [15, 16] were not included in the review, and none of the studies reviewed included hormone measures [7].

We found that including a urinary estrogen metabolite in addition to mother’s report further improved the discrimination of breast onset (TS2+). Investigators planning to assess breast onset may want to consider incorporating estrogen biomarkers in their pubertal assessment because including E1G in our study improved the AUC by up to 0.11. However, estrogen alone does not fully capture clinically assessed breast onset, because there is substantial overlap in hormone-level distributions between stages of breast development, as we and others have shown [15, 34, 35]. Prior studies proposed that relatively weak correlations between clinician’s and mother’s reports of breast development and hormone levels may be due to accounting inadequately for menstrual cycle phase of biospecimen collection [15, 34]. However, menstrual cycle day is not a source of variation in our study, because all estrogen measures were taken from premenarcheal girls. Rather, some of the wide variation in E1G in TS2 across all assessment methods may be explained by the inclusion of girls with transient thelarche as well as girls with permanent thelarche in this group (*see* Fig. 1). Girls with transient thelarche (i.e., the appearance of breast onset that regresses and appears again) have lower hormone profiles than girls with permanent thelarche [36]. Whether to include estrogen measurement in a study design of pubertal development depends on the overall intent of the study. For example, for studies where there is interest in identifying breast onset as a period of breast cancer susceptibility, transient thelarche may be sufficiently captured by PDS assessment method (and estrogen measurement is not necessary), because the appearance of breast tissue marks a period of cell proliferation and rapid breast tissue development.

Because we have shown that estimates of age at breast onset, typically the first sign of pubertal development, differ depending on the assessment method, the degree of misclassification by each method has implications when using pubertal onset as either an outcome or a parameter to define the pubertal window of susceptibility. A focus of pediatric research since the 1990s has been determining whether there is a secular decline in the age at breast onset [9]. Although it appears that there has been a decline in the age at breast onset [5, 6, 37], one of the main critiques of early studies of pubertal timing in the United States was that even the gold standard, clinical breast Tanner staging, was limited if palpation was not performed [38]. There was concern that physical examination without palpation could not accurately distinguish true TS2 from lipomastia caused by obesity. In our study, two of the LEGACY sites used clinically trained providers to measure TS with palpation when necessary to rule out misclassification due to lipomastia. Measuring clinical TS may also still be extremely useful in young girls because the specificity of PDS is still very low in mothers of young girls. Until the assessment of breast onset is standardized, one way to draw comparisons across future studies is to assess breast onset using all three methods in a subset of the study population, particularly in young girls, so that final estimates can be adjusted for measurement error [20]. For studies that cannot implement all three methods, the measurement error estimates from our study can be used, as long as the limitations of our study are considered. Although we did not observe major differences based on breast cancer family history, our enriched study based on half of the participants having a breast cancer family history may not be generalizable to other populations. Our study also does not address whether mother’s report of breast onset would be a reliable measure in other countries; however, mothers living in selected cultures may prefer the PDS because it does use pictures of breasts.

Ultimately, assessing breast onset accurately in easily scalable ways is essential to advancing the understanding of how early life influences breast cancer risk, as well as understanding pubertal trends and their health impacts more broadly. Early breast onset (< 10 years old compared with 11–12 years) is associated with a 23% increased risk of breast cancer [4]. We found a 2- to 8-month difference in the age at onset, depending on whether mother’s report of breast onset was assesed by PDS or SMS. Considering that a 1-month delay in age at breast onset is related to a 1.6% decrease in breast cancer risk [4], it is important to consider the expected effect size of the association in relation to the size of measurement error. For example, in a recent longitudinal study of breast onset assessed by annual clinical TS, obesity (BMI > 95th percentile) was associated with an 8.4-month acceleration in median age of breast onset compared with nonobese U.S. girls (50th to < 85th percentile) [5]. The median age of breast onset was also 6.7 months earlier in this population of girls born between 1996 and 2002 compared with girls born between 1980 and 1990 [5, 6]. Both of these studies assessed breast onset using an annual clinical assessment of breast onset for the majority of their participants. However, in the study by Biro et al., only a subset of girls was assessed semiannually, and the authors explained that semiannual vs annual assessment could account for a 3- to 4-month difference in the age of breast onset between the studies [5]. A clear advantage of using PDS over clinical TS to assess breast onset are that (1) it can be implemented more frequently and in a more cost-effective and scalable manner, and (2) it may yield tighter estimates of median age of breast onset, especially for exposures of interest that may have associations of smaller magnitude than body size or secular time.

## Conclusions

Mother’s report of breast onset using PDS is a viable alternative to mother’s report using SMS for large-scale epidemiological studies of breast onset. The method used for breast onset assessment alters the estimates of median age at onset, which has implications for studies of pubertal onset and studies of breast cancer concerned with pubertal timing. Including biomarkers related to breast onset, such as urinary estrogen, can improve the accuracy of pubertal assessments.

## Additional file

Additional file 1: **Table S1.** Study characteristics in the cohort and subsets. (DOCX 14 kb)

## Abbreviations

BMI: Body mass index
CDC: Centers for Disease Control and Prevention
E1G: Estrone 1-glucuronide
EIA: Enzyme immunoassay
PDS: Pubertal Development Scale
SMS: Sexual Maturation Scale
TS: Tanner stage
