# Supplementary material for: Comparison of methods to assess onset of breast development in the LEGACY Girls Study: methodological considerations for studies of breast cancer
Source: Breast Cancer Res. 2018 Apr 18;20:33. doi: 10.1186/s13058-018-0943-9 (PMC5907380; doi:10.1186/s13058-018-0943-9)
Supplement: Supplementary file 1 — Table S1. Study characteristics in the cohort and subsets. (DOCX 14 kb) [file 13058_2018_943_MOESM1_ESM.docx]

Supplemental Table. Study Characteristics in the Cohort and Subsets

|  | All (n=1022) | Subset with Clinical TS (n=282) | Subset with Clinical TS and E1G (n=153) |
| --- | --- | --- | --- |
| Age |  |  |  |
| Daughter Age <10 | 49% | 61% | 71% |
| Daughter Age ≥10 | 51% | 39% | 29% |
| Family History |  |  |  |
| Positive | 52% | 45% | 60% |
| Negative | 48% | 55% | 40% |
| BMI Percentile |  |  |  |
| BMI≥85% percentile | 19% | 18% | 16% |
| BMI<85% percentile | 81% | 82% | 84% |
| Race/Ethnicity |  |  |  |
| White Non-Hispanic | 62% | 67% | 67% |
| Hispanic | 19% | 21% | 22% |
| Other (Asian, black, other) | 19% | 12% | 11% |
| PDS |  |  |  |
| 1 | 46% | 56% | 63% |
| 2 | 22% | 19% | 23% |
| 3 | 29% | 24% | 14% |
| 4 | 3% | 1% | 0% |
| SMS |  |  |  |
| 1 | 55% | 65% | 76% |
| 2 | 16% | 14% | 13% |
| 3 | 17% | 11% | 8% |
| 4 | 12% | 10% | 3% |
| Clinical TS |  |  |  |
| 1 |  | 56% | 67% |
| 2 |  | 21% | 24% |
| 3 |  | 13% | 7% |
| 4 |  | 10% | 2% |
